# Childhood trauma and suicidal ideation among Chinese university students: the mediating effect of Internet addiction and school bullying victimisation

**DOI:** 10.1017/S2045796020000682

**Published:** 2020-08-10

**Authors:** Li Lu, ShengYan Jian, Min Dong, Jie Gao, TianTian Zhang, XueQin Chen, YuFang Zhang, HongYi Shen, HongRu Chen, XiangYun Gai, Shou Liu

**Affiliations:** 1 Department of Public Health, Medical College, Qinghai University, Xining, Qinghai, China; 2 Team IETO, Bordeaux Population Health Research Center, UMR U1219, INSERM, Université de Bordeaux, Bordeaux, France; 3 Qinghai Provincial People's Hospital, Xining, Qinghai, China; 4 Guangdong Mental Health Center, Guangdong Provincial People's Hospital, Guangdong Academy of Medical Sciences, Guangzhou, Guangdong, China; 5 Department of Clinical Medicine, Qinghai Institute Of Health Sciences, Xining, Qinghai, China; 6 Department of Public Education, Xining Urban Vocational & Technical College, Xining, Qinghai, China; 7 School of Pharmacy, Qinghai Nationalities University, Xining, Qinghai, China

**Keywords:** Childhood trauma, China, Internet addiction, suicidal ideation, school bullying victimisation, university students

## Abstract

**Aims:**

The factors associated with suicidal ideation among adolescents have been extensively characterised, but the mechanisms underlying the complexities of the relationship between experiences of childhood trauma and suicidal ideation have been less studied. This study examined the direct effect of childhood trauma on suicidal ideation on the one hand and whether school bullying victimisation and Internet addiction mediate the association between childhood trauma and suicidal ideation on the other hand.

**Methods:**

This school-based mental health survey was carried out in Qinghai Province in Northwest China in December 2019. We employed standardised questionnaires to collect sociodemographic and target mental health outcomes. Hierarchical multiple logistic regression and structural equation modelling were performed for the data analyses.

**Results:**

This study included 5864 university students. The prevalence of lifetime suicidal ideation and Internet addiction were 34.7% and 21.4%, respectively. Overall, 16.4% and 11.4% of participants reported experiences of childhood trauma and school bullying victimisation, respectively. There were direct effects of childhood trauma, school bullying victimisation and Internet addiction on suicidal ideation. The total effect of childhood trauma on suicidal ideation was 0.201 (*p* < 0.001). School bullying victimisation and Internet addiction mediated the relationship between childhood trauma and suicidal ideation. Internet addiction played a mediating role between school bullying and suicidal ideation.

**Conclusions:**

Childhood trauma had both direct and indirect effects on suicidal ideation; these effects were mediated by school bullying victimisation and Internet addiction in Chinese university students. Elucidating these relationships will therefore be useful in developing and implementing more targeted interventions and strategies to improve the mental well-being of Chinese university students.

## Introduction

Suicide occurs across the lifespan and is the second-leading cause of death among 15- to 29-year-olds worldwide (World Health Organization (WHO), 2016). Suicide-related issues among Chinese children and adolescents are repeatedly emphasised (Chen *et al*., 2018; Guo *et al*., 2018), such as suicidal ideation which is a significant risk factor for suicidal attempts and death (Barzilay *et al*., 2017; Kwok *et al*., 2019). The existing body of research has demonstrated that the prevalence of suicidal ideation among Chinese college students ranges from 1.24% to 26.00% (Li *et al*., 2014). Socioeconomic adversity, adverse childhood events, bullying victimisation, substance abuse and psychological problems are identifiable predictors that contribute to the development of adolescent suicidal ideation (Barzilay *et al*., 2017; Tang *et al*., 2018; Kwok *et al*., 2019; Kim and Chun, 2020; Wang *et al*., 2020).

Childhood trauma has been established as the principal predictor of lifetime DSM-IV disorders (Kessler *et al*., 2010; Kircaburun *et al*., 2019), and evidence has successively suggested that the likelihood of suicidal ideation among students increased as the probability of childhood trauma experienced increased (Jeon *et al*., 2009; Clements-Nolle *et al*., 2018). For instance, a longitudinal study in the USA found that the accumulation of adverse childhood experiences increased the odds of suicidal ideation in adulthood (Thompson *et al*., 2019). The mechanisms regarding the complexities of the relationship between experiences of childhood trauma and suicidal ideation draw increasing research attention.

In addition, whether an offender or a victim, youth who experienced bullying had more suicidal ideations than those who had not experienced such patterns of peer aggression (Hinduja and Patchin, 2010). One Chinese study with a sample of 4034 university students also revealed an association between bullying experiences during primary and secondary school and a higher risk of suicidal ideation in young adulthood (Wang *et al*., 2020). Internet addiction among university students became a matter of concern along with the dramatically increased Internet use. It is undeniable that the Internet benefits users to some extent, while it produces several maladaptive and detrimental consequences, such as poor quality of life and suicidal ideation (Guo *et al*., 2018; Lu *et al*., 2018). The mitigation of school bullying requires a dedicated team of families, educators, health-care professionals and policymakers (Srabstein and Leventhal, 2010; Shayo and Lawala, 2019); these individuals are also critical in helping youth affected by Internet addiction.

The mediating effects of several factors in the relationship between childhood trauma and suicidal ideation were determined, such as gratitude and interpersonal difficulties in the form of social inhibition, emotion dysregulation, negative schema and rumination (Cui *et al*., 2019; Kwok *et al*., 2019; Lemaigre and Taylor, 2019). This leads us to suspect that Internet addiction and school bullying could be mediating factors of interest between experiences of childhood trauma and suicidal ideation. Evidence surrounding the mediating roles of school bullying and Internet addiction in the relationship between childhood trauma and suicide ideation has not yet been probed, specifically at the university student level, which thus requires further clarification. Furthermore, examining the previously described speculation will help disentangle the underlying relationships and yield beneficial information for targeting prevention efforts.

Therefore, the primary objective of the present study is to describe the prevalence of childhood trauma, suicidal ideation, school bullying victimisation and Internet addiction in a population-based sample of Chinese university students in Qinghai-Tibetan areas. A secondary objective is to investigate the degree to which the direct association between childhood trauma and suicidal ideation is valid and to examine the mediating roles of school bullying victimisation and Internet addiction in the relationship between childhood trauma and suicidal ideation using structural equation modelling. Building upon previous research, we hypothesised that childhood trauma would be directly and indirectly related to suicidal ideation via school bullying victimisation and Internet addiction.

## Methods

### Study design and data collection

This large-scale school-based mental health survey was carried out in Qinghai Province in Northwest China in December 2019. A multistage-stratified cluster sampling method was used to recruit the participants. There are 12 universities or colleges in Qinghai Province. First, a stratified sampling method was used to select universities by taking the affiliation levels and classifications of the universities as the indicators. A total of four universities were selected, including Qinghai University (one of the national ‘211 Project’ universities), Qinghai Nationalities University (a provincial-level ethnic undergraduate university), Qinghai Institute Of Health Sciences (a provincial-level industry supervisor undergraduate college) and Xining Urban Vocational & Technical College (a municipal vocational college). In each university or college, a stratified (according to the majors) random sampling method was used to select the classes, and cluster sampling was then used in each class.

Questionnaires were distributed to participants and collected after completion by our study investigators who were uniformly trained prior to the on-site survey. Students who were fully enrolled in the universities were included. A total of 6500 questionnaires were distributed, and 6200 questionnaires were returned, yielding a response rate of 95.4%. Students from Qinghai University, Qinghai Nationalities University, Qinghai Institute Of Health Sciences and Xining Urban Vocational & Technical College accounted for 30.0%, 27.5%, 26.9% and 15.7% of the sample, respectively. Finally, data from 5864 participants were analysed in this study after cases with ⩾ 20% missing data were deleted.

The Ethics Committee of the Medical College of Qinghai University approved the study protocol. The survey process followed the principles of anonymity and voluntariness, and all university students involved in this survey provided the informed consent. We followed the Strengthening the Reporting of Observational Studies in Epidemiology (STROBE) guidelines to report this study (Von Elm *et al*., 2007).

### Assessment

#### Basic characteristics

Basic sociodemographic and clinical information, including age (years), sex (male/female), place of residence prior to entering the university (non-plateau/plateau area), ethnicity (Han/others), self-perceived family economic level (rich/general/poor), only-child status (no/yes), self-perceived weight (underweight/normal/overweight), self-perceived health (good/general/bad), whether in a dating relationship (no/yes) and relationships with classmates, teachers and family (poor/fair/good), was collected.

#### Suicidal ideation

Suicidal ideation (SI) was assessed using the fourth and fifth items of the Beck Scale for Suicidal Ideation (BSS) (Beck *et al*., 1979), which is widely used as a self-report screening tool (Brown *et al*., 2000; van Spijker *et al*., 2010). Lifetime SI was considered if there was at least one positive response to the questions.

#### Childhood trauma

Childhood trauma was assessed by the following question, ‘Have you suffered severe psychological trauma or significant life adversity before the age of 16?’ Possible answers were no or yes.

#### Mediating variables

The Internet Addiction Test (IAT), which has satisfactory psychometric properties (Cronbach's *α*: 0.713) (Young, 1998, 2008), has been widely validated among countries (Lam *et al*., 2009; Young, 2013), and its Chinese version was used to examine the presence and severity of IA in our study. A total score of ⩾ 50 indicated moderate and severe dependence on the Internet and was defined as ‘having IA’ (Young, 2008), which has been used in previous studies (Yoo *et al*., 2004; Karacic and Oreskovic, 2017; Lu *et al*., 2018). School bullying victimisation was assessed by a ‘yes/no’ question: ‘In the past year, have you been bullied or threatened by others at school (for example, other students tease you on purpose or give you nicknames that you do not like; classmates have deliberately left you out during class breaks or upset you; you have been beaten by others; other students have urged you to do something for them even if you do not want to, etc.)?’

### Data analyses

The sociodemographic and clinical characteristics were described with the number (*n*) and percentage (%) or the mean and standard deviation (s.d.), as appropriate. Hierarchical multiple logistic regression was carried out to examine the associations between experiences of childhood trauma and suicidal ideation. In step 1, the model was unadjusted by setting suicidal ideation as the dependent variable and childhood trauma as the independent variable. In step 2, adjustments were made for age (years), sex, place of residence prior to entering university, ethnicity, self-perceived family economic level, only-child status, self-perceived weight, self-perceived health status, whether in a dating relationship, relationships with classmates, relationships with teachers or relationships with family. In step 3, school bullying victimisation was added, and Internet addiction was added in the last step. At each step, the R^2^ change (Δ*R*^2^) was used to indicate the predictive power of each group of predictor(s) when adjustments were made for previous predictor(s). A *post hoc* analysis was performed by reversing steps 3 and 4. The results were expressed with odds ratios (ORs) and their 95% confidence intervals (CIs).

We performed a structural equation model (SEM) to evaluate the hypothesis of the mediating effects of Internet addiction and school bullying victimisation in the relationship between childhood trauma and suicide ideation. Sociodemographic and clinical characteristics that showed statistical significance in step 4 in hierarchical multiple logistic regression were adjusted in the SEM. We used the R lavaan package (Rosseel, 2012), and a comparative fit index (CFI) ⩾ 0.90, a Tucker–Lewis index (TLI) ⩾ 0.95, a root mean square error of approximation (RMSEA) < 0.08 and a standardised root mean square residual (SRMR) < 0.08 indicate satisfactory goodness of fit (Hooper *et al*., 2008; Kline, 2015). In all models, only those cases without missing data were analysed. All data were analysed with RStudio software (Version 1.2.1335, ©2009–2019 RStudio, Inc.), with a significant *α* threshold of 0.05 (two tailed) .

## Results

### Sample characteristics

A total of 5864 university students with an average age of 19.9 years (s.d. = 1.52) were included in this study. Among the participants, 62.4% were (3657) female, 44.8% (2629) were of Han ethnicity and 79.4% (4656) lived in high-altitude areas prior to entering the university. Table 1 shows the basic characteristics of the participants.

**Table 1.** Basic characteristics of the participants

Variables	Categories	SI (*n* = 2037)	Overall (*n* = 5864)
Age years (mean ± s.d.)		19.8 ± 1.53	19.9 ± 1.52
Sex (*N* = 5750)	Female	1374 (67.5%)	3657 (62.4%)
	Male	631 (31.0%)	2093 (35.7%)
Place of residence prior to entering the university (*N* = 5659)	Non plateau	343 (16.8%)	1003 (17.1%)
	Plateau	1626 (79.8%)	4656 (79.4%)
Ethnicity (*N* = 5795)	Han	997 (48.9%)	2629 (44.8%)
	Other	1019 (50.0%)	3166 (54.0%)
Family economy (*N* = 5844)	Rich	106 (5.2%)	352 (6.0%)
	General	1512 (74.2%)	4449 (75.9%)
	Poor	413 (20.3%)	1043 (17.8%)
Only-child status (*N* = 5536)	No	1487 (73.0%)	4350 (74.2%)
	Yes	444 (21.8%)	1186 (20.2%)
Self-perceived weight (*N* = 5849)	Underweight	264 (13.0%)	751 (12.8%)
	Normal	1110 (54.5%)	3535 (60.3%)
	Overweight	660 (32.4%)	1563 (26.7%)
Self-perceived health (*N* = 5848)	Good	640 (31.4%)	2692 (45.9%)
	General	1265 (62.1%)	2954 (50.4%)
	Bad	126 (6.2%)	202 (3.4%)
Whether in a dating relationship (*N* = 5811)	No	1334 (65.5%)	3742 (63.8%)
	Yes	687 (33.7%)	2069 (35.3%)
Relationship with classmates (*N* = 5856)	Good	757 (37.2%)	2807 (47.9%)
	General	1224 (60.1%)	2962 (50.5%)
	Poor	53 (2.6%)	87 (1.5%)
Relationship with teachers (*N* = 5852)	Good	552 (27.1%)	2183 (37.2%)
	General	1405 (69.0%)	3533 (60.2%)
	Poor	78 (3.8%)	136 (2.3%)
Relationship with family (*N* = 5828)	Good	1569 (77.0%)	5055 (86.2%)
	General	421 (20.7%)	716 (12.2%)
	Poor	33 (1.6%)	57 (1.0%)
School bullying victimisation (*N* = 5832)	No	1699 (83.4%)	5170 (88.2%)
	Yes	320 (15.7%)	662 (11.3%)
Internet addiction (*N* = 5757)	No	1393 (68.4%)	4624 (78.9%)
	Yes	640 (31.4%)	1233 (21.0%)
Experiences of childhood trauma (*N* = 5718)	No	1459 (71.6%)	4782 (81.5%)
	Yes	526 (25.8%)	936 (16.0%)

The prevalence of lifetime suicidal ideation and Internet addiction were 34.7% (2037/5864; 95% CI 33.5–36.0%) and 21.4% (1233/5757; 95% CI 20.4–22.5%), respectively. Overall, 16.4% (936/5718; 95% CI 15.4–17.4%) and 11.4% (662/5832; 95% CI 10.5–12.2%) of university students reported experiences of childhood trauma and school bullying, respectively.

### Hierarchical regression analyses

Table 2 displays the results of hierarchical regression analyses. In total, basic sociodemographic and clinical indicators accounted for 14.8% of the variance in the outcomes beyond the effects of experiences of childhood trauma (step 2) (adjusted *R*^2^ = 0.201, Δ*R*^2^ = 0.148). School bullying victimisation, tested in step 3, captured an additional 0.8% of variance in suicidal ideation beyond the effects of basic sociodemographic and clinical factors and the experiences of childhood trauma (adjusted *R*^2^ = 0.209, Δ*R*^2^ = 0.008). When Internet addiction was added in the last step, it yielded an additional 0.8% of the variance (adjusted *R*^2^ = 0.217, Δ*R*^2^ = 0.008, *p* < 0.001), showing that experiences of childhood trauma (OR = 2.13, 95% CI 1.80–2.52), Internet addiction (OR = 1.87, 95% CI 1.61–2.17) and school bullying victimisation (OR = 1.58, 95% CI 1.29–1.92) were positively associated with suicidal ideation. When we reversed the order of entry in the regression model, entering Internet addiction in the third step, school bullying victimisation predicted suicide ideation over and above Internet addiction in the fourth step (Δ*R*^2^ = 0.005, *p* < 0.001).

**Table 2.** Results of hierarchical regression analyses in Chinese university students

Variables	Step 1	Step 2	Step 3	Step 4
Age years		0.91 (0.87–0.94)***	0.90 (0.87–0.94)***	0.90 (0.87–0.94)***
Sex (male)		0.75 (0.65–0.86)***	0.74 (0.65–0.85)***	0.73 (0.63–0.84)***
Place of residence prior to entering the university (plateau)		1.13 (0.96–1.34)	1.14 (0.97–1.35)	1.16 (0.98–1.38)
Ethnicity (other)		0.91 (0.80–1.04)	0.91 (0.80–1.04)	0.89 (0.78–1.01)
Family economy				
Rich		0.71 (0.52–0.96)*	0.72 (0.53–0.97)*	0.72 (0.53–0.97)*
Poor		1.05 (0.89–1.23)	1.05 (0.89–1.23)	1.03 (0.88–1.22)
Only-child status (yes)		1.05 (0.90–1.23)	1.05 (0.90–1.23)	1.07 (0.91–1.24)
Self-perceived weight				
Underweight		1.08 (0.88–1.31)	1.06 (0.87–1.29)	1.04 (0.85–1.27)
Overweight		1.26 (1.09–1.45)**	1.24 (1.08–1.43)**	1.19 (1.03–1.38)*
Self-perceived health				
General		0.50 (0.36–0.69)***	0.51 (0.36–0.71)***	0.53 (0.38–0.73)***
Good		0.26 (0.19–0.36)***	0.27 (0.19–0.37)***	0.28 (0.20–0.39)***
Whether in a dating relationship (yes)		0.87 (0.76–0.99)*	0.86 (0.76–0.99)*	0.87 (0.76–0.99)*
Relationship with classmates				
General		0.75 (0.44–1.25)	0.78 (0.46–1.31)	0.83 (0.49–1.41)
Good		0.59 (0.35–0.99)*	0.63 (0.38–1.06)	0.69 (0.40–1.18)
Relationship with teachers				
General		0.77 (0.50–1.18)	0.77 (0.51–1.19)	0.84 (0.55–1.30)
Good		0.61 (0.39–0.95)*	0.61 (0.39–0.96)*	0.69 (0.44–1.08)
Relationship with family				
General		1.55 (0.81–2.96)	1.63 (0.84–3.17)	1.60 (0.82–3.13)
Good		0.72 (0.38–1.36)	0.76 (0.40–1.46)	0.76 (0.39–1.47)
School bullying victimisation (yes)			1.72 (1.42–2.10)***	1.58 (1.29–1.92)***
Internet addiction (yes)				1.87 (1.61–2.17)***
Experiences of childhood trauma (yes)	2.91 (2.53–3.37)***	2.25 (1.91–2.66)***	2.17 (1.83–2.56)***	2.13 (1.80–2.52)***
Adjusted *R*^2^	0.053	0.201	0.209	0.217
*ΔR*^2^		0.148	0.008	0.008

Step 1 (*N* = 5637): unadjusted.

Step 2 (*N* = 5040): adjusted for age years, sex, place of residence prior to entering the university, ethnicity, self-perceived family economic level, only-child status, self-perceived weight, self-perceived health status, whether in a dating relationship, relationship with classmates, relationship with teachers, relationship with family.

Step 3 (*N* = 5017): Model 2 variables + school bullying victimisation.

Step 4 (*N* = 5017): Model 3 variables + Internet addiction.

*p < 0.05, **p < 0.01, ***p < 0.001 (two-tailed).

### Structural equation modelling

**Fig. 1.**
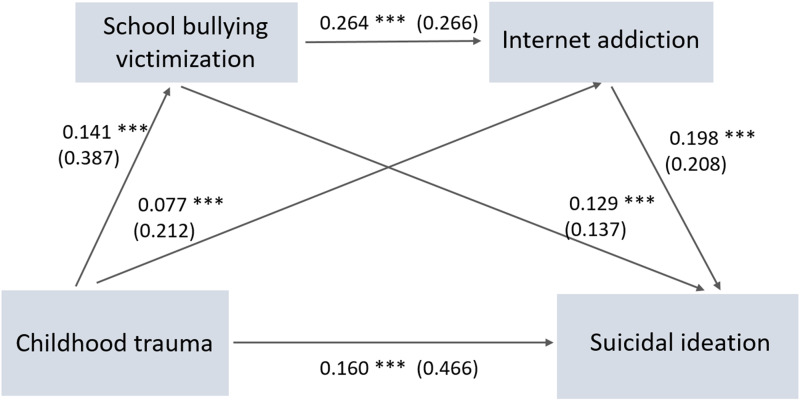
Final model with the standardised coefficients (*β*), and unstandardised coefficients (*β*) presented in the parentheses (*N* = 5420). ***p < 0.001. CFI = 1.000; TLI = 1.000; RMSEA = 0; SRMR = 0.006.

Figure 1 shows the results of structural equation modelling. There were direct effects of childhood trauma (*β* = 0.160, *p* < 0.001), school bullying victimisation (*β* = 0.129, *p* < 0.001) and Internet addiction (*β* = 0.198, *p* < 0.001) on suicidal ideation. The total effect of childhood trauma on suicidal ideation was 0.201 (*p* < 0.001). The final SEM also revealed the mediating effects of school bullying victimisation and Internet addiction on the association between childhood trauma and suicidal ideation (*β* = 0.018, *p* < 0.001 and *β* = 0.015, *p* < 0.001, respectively). School bullying victimisation also had an indirect effect on suicidal ideation which was mediated by Internet addiction (*β* = 0.052, *p* < 0.001). Goodness-of-fit indices (i.e. CFI = 1.000; TLI = 1.000; RMSEA = 0; SRMR = 0.006) indicated satisfactory fit of the SEM.

## Discussion

This study, based on a sample of 5864 university students from parts of the Chinese Tibetan Plateau (i.e. Qinghai Province), allowed us to identify the following: (1) our mental health problems of interest were common among Chinese university students; (2) childhood trauma, school bullying victimisation and Internet addiction had associations with suicidal ideation among the population of interest; (3) there were indirect effects of childhood trauma on suicidal ideation, which were mediated by school bullying victimisation and Internet addiction; and (4) Internet addiction played a mediating role in the relationship between school bullying victimisation and suicidal ideation.

At present, suicidal ideation among adolescents is widely concerned around the world (Mortier *et al*., 2018). The lifetime prevalence of suicidal ideation among our participants (34.7%; 95% CI 33.5–36.0%) was approximately 1.5 times that of the worldwide prevalence among college students (22.3%, 95% CI 19.5–25.3%) estimated in one meta-analysis (Mortier *et al*., 2018). Our figure was also greater than those presented in other Chinese surveys in the same targeted population, such as 7.3% in a study of 5972 university students from Wuhan, Hubei Province (Tang *et al*., 2018) and 9.9% in a study of 4034 university students from Anhui Province (Wang *et al*., 2020). These discrepancies may be partially attributed to the different assessment instruments used as well as the evaluated durations of suicidal ideation, for example, the other two Chinese studies assessed the prevalence in the last 12 and 6 months, respectively, while we evaluated the lifetime prevalence. In addition, sociodemographic differences (Nock *et al*., 2008) and disparities in college-specific factors (Eisenberg *et al*., 2013) may simultaneously play potential roles.

After adjustments were made for the control variables, hierarchical regression models indicated that childhood trauma, school bullying victimisation and Internet addiction increased the likelihood of having suicidal ideation. We thus conducted SEM by adjusting for sociodemographic factors, personal health factors and dating status, and we identified the direct effect as well as the indirect effect of childhood trauma on suicidal ideation, the latter of which was mediated by school bullying victimisation. Consistently, the direct effect of childhood trauma on suicidal ideation was demonstrated in another Chinese study including 922 freshmen (Shi *et al*., 2020). A moderately significant degree of correlation between suicidal ideation and exposure to early trauma was also identified among Indian college students (Singh *et al*., 2012). In terms of the role of school bullying victimisation, the strong relationship between adverse childhood experiences and the probability of on-campus victimisation was identified among high school students (Forster *et al*., 2020), and the latter can independently predict the likelihood of suicidal ideation among school adolescents (Barzilay *et al*., 2017; Wang *et al*., 2018; Shayo and Lawala, 2019), which can support our finding.

Internet addiction also played a mediating role in the relationship between childhood trauma and suicidal ideation. Childhood trauma and its subtypes, such as emotional, physical and sexual abuse, were reported as factors associated with Internet addiction or Internet gaming disorders in different populations (Dalbudak *et al*., 2014; Schimmenti *et al*., 2014; Kircaburun *et al*., 2019; Shi *et al*., 2020). Internet use could be a more popular coping strategy to avoid concentrating on experiences of trauma or bullying and stressful life events or to elevate mood (Park *et al*., 2013; Shi *et al*., 2020). For example, students with childhood traumatic experiences or being bullied would prefer to share their experiences and obtain comfort through communicating with netizens from social networking platforms instead of the familiar individuals in the real world, especially those with borderline personality features (Dalbudak *et al*., 2014), lower social support (Karaer and Akdemir, 2019) or increased loneliness (AJ *et al*., 2014), etc., which also explained the relationship between school bullying and Internet addiction. Furthermore, in line with our results, one survey in China (Guo *et al*., 2018) and another in South Korea (Park *et al*., 2013) with 20 895 and 795 high school students, respectively, both suggested the direct effects of Internet addiction on suicidal ideation. Mobile phones are one of the major modes of access to the Internet, and adolescents' dependence on their phones is also a predictor of suicidal ideation (Chen *et al*., 2020). Consequently, childhood trauma can be indirectly linked with suicidal ideation through Internet addiction. However, only a few relevant studies concerning the above findings that were available and focused on university students, and our study extended this literature.

The findings underscore the importance and necessity of implementing suicide intervention strategies and preventing adverse childhood events and invisible or visible on-campus bullying and Internet addiction. Professional levels of psychological counselling and guidance, mental health education courses, campus safety management and other interventions should be considered and practically implemented (Jimerson and Furlong, 2006; Chen *et al*., 2018; Strom *et al*., 2018). However, there were several limitations that should be noted. First, the cross-sectional nature of our study makes it impossible to capture the causality, and future research might benefit from longitudinal studies. Second, the potential recall bias cannot be avoided which could produce the potential estimation errors. Additionally, the mental health outcomes of interest were assessed using self-reported screening questionnaires or questions rather than clinical diagnostics, which could be less helpful in clinical significance. However, our results have still provided evidence from epidemiological and screening perspectives. Finally, the target population volunteered to participate in this survey, and approximately two out of every three participants in our sample were female university students. Therefore, the study results may not be generalisable to all Chinese university students.

In conclusion, this study extended the findings of previous literature by elucidating the direct effects of childhood trauma, school bullying victimisation and Internet addiction on suicidal ideation among university students, as well as the mediating roles of school bullying victimisation and Internet addiction in the relationship between childhood trauma and suicidal ideation. Integrally targeted interventions and strategies that can eliminate and alleviate school bullying events, Internet addiction and the influences of childhood trauma should be developed and implemented to reduce the risk of suicidal ideation and improve the comprehensive mental well-being of Chinese university students.

## Data Availability

For more information, email to liushou2004@aliyun.com.
